# Neural Correlates of Conflict Control on Facial Expressions with A Flanker Paradigm

**DOI:** 10.1371/journal.pone.0069683

**Published:** 2013-07-24

**Authors:** Tongran Liu, Tong Xiao, Jian-Nong Shi

**Affiliations:** 1 Key Laboratory of Behavioral Science, Institute of Psychology, Chinese Academy of Sciences, Beijing, China; 2 Natural Language Processing Laboratory, Northeastern University, Liaoning, China; 3 Department of Learning and Philosophy, Aalborg University, Aalborg, Denmark; University College London, United Kingdom

## Abstract

Conflict control is an important cognitive control ability and it is also crucial for human beings to execute conflict control on affective information. To address the neural correlates of cognitive control on affective conflicts, the present study recorded event-related potentials (ERPs) during a revised Eriksen Flanker Task. Participants were required to indicate the valence of the central target expression while ignoring the flanker expressions in the affective congruent condition, affective incongruent condition and neutral condition (target expressions flanked by scramble blocks). Behavioral results manifested that participants exhibited faster response speed in identifying neutral target face when it was flanked by neutral distractors than by happy distractors. Electrophysiological results showed that happy target expression induced larger N2 amplitude when flanked by sad distractors than by happy distractors and scramble blocks during the conflict monitoring processing. During the attentional control processing, happy target expression induced faster P3 response when it was flanked by happy distractors than by sad distractors, and sad target expression evoked larger P3 amplitude when it was flanked by happy distractors comparing with sad distractors. Taken together, the current findings of temporal dynamic of brain activity during cognitive control on affective conflicts shed light on the essential relationship between cognitive control and affective information processing.

## Introduction

Conflict control refers to the ability of monitoring the conflicts within perceptual inputs or between the required response and the preferred response, and then executes attentional control upon the conflicts [1]. Botvinick et al. [2] proposes the cognitive control theory and regards that the specific brain structure of anterior cingulated cortex (ACC) is strongly activated when conflicts occur in several cognitive circumstances that involved response override, or decisions among comparable responses, or committing errors. Some recent studies put forward that it is essential to explore how individuals attend to and execute conflict control on the perceptual inputs with affective information (emotion-laden words, facial expressions) [3,4].

A behavioral flanker task has been implemented to investigate how participants execute conflict control on target facial expressions when they are flanked by identical expressions (congruent condition) or different expressions (incongruent condition) [5], and it is observed that participants exhibit faster response speed when the distractor expressions are identical with the target expression, and the flanker effect is smaller when the targets are negative faces compared with positive faces. The phenomenon is explained as that negative target faces narrow the focus of attention, while positive target faces broaden the focus of attention [5,6]. In the same vein, Ochsner and colleagues [7] utilized functional magnetic resonance imaging (fMRI) technology to investigate the brain structures related with conflict control on affective information, and they asked participants to indicate the valence of a central target word while ignoring the distractor words. It was reported that participants reacted slower in the affective incongruent condition than in the congruent condition, meanwhile the bilateral dorsal ACC were more activated in the incongruent condition. However, until now, the temporal dynamic of conflict control processing on affective inputs is still unknown, hence, people still cannot tell how brain executes conflict control upon the affective conflicts within different temporal courses.

The event-related potential technique (ERP) can record electrophysiological signals in human brain with high temporal resolution (millisecond level), and the existed studies on conflict control upon non-affective stimuli indicate that when the conflict situations occur, a negative component peaking at about 200 ms (N2) is evoked with the neural generator locating at the ACC and reflects the neural processing of initial conflict monitoring [1,8,9]. The N2 amplitude correlates with the levels of conflict situations, and the higher conflict condition (the incongruent condition) induces stronger N2 activation than the lower conflict condition (the congruent condition) [9–11]. Also, a positive component (P3) peaking at 300-600 ms range is evoked after conflict onset and is regarded as relating with attentional control on the conflicts, and more conflicts elicit larger P3 activation [12,13].

The main goal of current study is to investigate the temporal dynamics of conflict control on affective information with the aid of ERP technique by utilizing a revised affective flanker task. In the present flanker task, real human faces with sad, happy and neutral expressions are adopted as the targets flanked by either sad, happy, neutral faces or scramble blocks as distractors. Our first hypothesis is that the affective incongruent condition induces larger N2 and P3 responses than the affective congruent condition and neutral condition (scramble block distractors). Also, according to the previous behavioral findings that the flanker effect is smaller when the targets are negative faces compared with positive faces [5], our second hypothesis is that the flanker effects with negative target expressions and positive expressions relate to separate neural processes. Our present study is to provide more evidences for the cortical processing of conflict control on affective information and to reveal the significant interplay between conflict control and facial expression perception.

## Results

### Behavioral results

Repeated measures ANOVAs were conducted to analyze reaction time and accuracy with 2 independent factors: target expressions (3 levels: neutral faces, happy faces, sad faces) and flanker distractors (4 levels: neutral faces, happy faces, sad faces and scrambled blocks). Analysis results showed that target expressions had significant main effect on accuracy (F(2,34)=5, *p*<0.03), and further multiple comparison test manifested that it took participants lower accuracy to percept sad target faces than neutral target faces (p<0.001). It was also observed that target expressions had significant main effect on reaction time (F(2,34)=4.9, *p*<0.03), and further multiple comparison test manifested that it took participants longer reaction time to percept sad target faces than neutral target faces (p<0.001). The interaction effect of target expressions × flanker expressions was also significant on reaction time (F(6,102)=2.97, *p*<0.05), and post hoc pair wise comparison indicated that it was faster to identify neutral target faces when they were flanked by neutral expressions than by happy distracter faces (*p*<0.05). The flanker effects induced by happy or sad target faces were not significant according to post hoc analyses (happy: F(6,102)=1.8, *p*>0.05; sad: F(6,102)=1.9, *p*>0.05). The mean reaction time and accuracy of behavioral responses were presented in Table 1.

**Table 1:** The mean reaction time (ms) and accuracy (from 0 to 1) of participants’ behavioral performances of conflict control processing on facial expression perception in the different conditions.

		Happy distractors	Sad distractors	Neutral distractors	Scramble blocks
Happy target faces	Accuracy	0.96±0.03	0.96±0.04	0.95±0.05	0.95±0.02
	Reaction time	578±57.5	581±55	581±50.5	581±52
Sad target faces	Accuracy	0.93±0.06	0.93±0.05	0.95±0.04	0.95±0.04
	Reaction time	596±66	596±56	599±59	592±57
Neutral target faces	Accuracy	0.97±0.03	0.97±0.03	0.96±0.04	0.96±0.05
	Reaction time	576±50	570±54	560±54	570±57

### ERP results

The peak latencies and amplitudes of N2 and P3 components in each condition were presented in Table 2.

**Table 2:** The mean latency (ms) and peak amplitude (µV) of N2 and P3 for the conflict control processing on facial expression perception in each condition.

		Happy distractors	Sad distractors	Neutral distractors	Scramble blocks
Happy target	N2 latency	244±33	243±35	243±35	242±34
	N2 amplitude	-2.7±2.7	-4.6±3.5	-3.4±3	-3.1±3
	P3 latency	453±45	480±55	479±55	460±54
	P3 amplitude	10.5±4.9	10.3±5.3	11.4±4.8	10±5
Sad target	N2 latency	234±34	243±33	249±37	242±35
	N2 amplitude	-3.2±2.8	-3.2±2.6	-3.2±3.2	-3.3±2.9
	P3 latency	474±57	466±52	464±45	451±56
	P3 amplitude	10.3±4	8.1±3.5	8.8±3.9	8.2±3.6
Neutral target	N2 latency	231±31	239±27	232±25	226±26
	N2 amplitude	-3.2±3.1	-2.8±2.8	-2.7±2.5	-3.3±2.7
	P3 latency	482±46	474±55	489±53	471±53
	P3 amplitude	11.6±4.7	11.5±3.6	10.8±3.7	10.5±4

The peak latencies and amplitudes of N2 component were subjected to repeated measures ANOVAs to analyze the conflict monitoring processing on affective conflicts with 3 within-subject factors: target expressions (3 levels: happy faces, sad faces, neutral faces), flankers (4 levels: happy faces, sad faces, neutral faces, scramble blocks) and hemisphere (3 levels: left hemisphere [clusters of F3 and FC3], mid-line [clusters of Fz and FCz], right hemisphere [clusters of F4 and FC4]) over frontal and central areas. Analysis results presented that target expressions and hemisphere had main effect on N2 latencies (target: (F(2,34)=4.6, *p*<0.05; hemisphere: F(2,34)=13.5, *p*<0.001), and further multiple comparisons showed that neutral target faces induced faster N2 responses than happy and sad targets (*p*<0.01) and right hemisphere evoked faster N2 response than left hemisphere and mid-line electrodes (*p*<0.005). For N2 amplitude, the interaction effect of target expressions × flanker expressions was significant on N2 amplitude (F(6,102)=3, *p*<0.05), and further post hoc pair-wise comparisons showed that happy target faces induced larger N2 amplitude when they were flanked by sad distractors than by happy distractors and scramble blocks (*p*<0.01).

**Figure 1:**
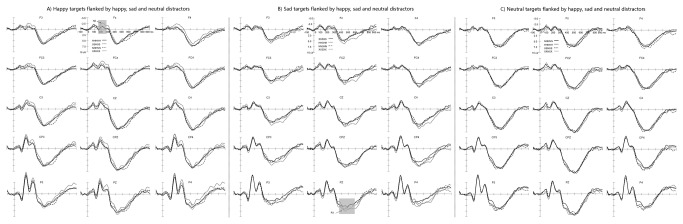
The N2 and P3 activation in each experimental condition. N2 amplitude in the SSHSS condition > the HHHHH condition and the SSHSS condition > the XXHXX condition. P3 amplitude in the HHSHH condition > the SSSSS condition.

The peak latencies and amplitudes of P3 component were subjected to repeated measures ANOVAs to study the attentional control processing on facial expression-related conflicts with 3 within-subject factors: target expressions (3 levels: happy faces, sad faces, neutral faces), flankers (4 levels: happy faces, sad faces, neutral faces, scramble blocks) and hemisphere (3 levels: left hemisphere [clusters of C3, CP3 and P3], mid-line [clusters of CZ, CPZ and PZ], right hemisphere [clusters of C4, CP4 and P4]) over central and parietal areas. For P3 peak latency, the main effect of flankers was significant (F(3,51)=2.7, *p*<0.05), and further pair comparison analysis showed that scramble blocks evoked faster P3 response than neutral distractors (*p*<0.05). The main effect of hemisphere was significant (F(2,34)=6.5, *p*<0.005), and further pair comparison analysis showed that right hemisphere induced faster P3 response than left hemisphere and mid-line electrode sites (*p*<0.05). The interaction effect of target expressions × flankers was significant on P3 latency (F(6,102)=2, *p*<0.05). Happy targets induced faster P3 responses when they were flanked by happy distractors than by sad distractors (*p*<0.05). Analysis on P3 amplitude manifested that target expressions had significant main effect on P3 amplitude (F(2,34)=13.2, *p*<0.001), and further multiple comparisons showed that sad targets induced smaller P3 amplitude than happy and neutral targets. The main effect of hemisphere was also significant on P3 amplitude (F(2,34)=14.7, *p*<0.001), and further pair comparisons showed that mid-line electrode sites induced larger P3 amplitudes than right and left hemisphere (*p*<0.001). The interaction effect of target expressions × flanker expressions was also significant on the P3 peak amplitude (F(6,102)=4.01, *p*<0.05), and the post hoc pair wise comparisons showed that sad target faces elicited larger P3 amplitude when they were flanked by happy distractors than sad distractors (*p*<0.005). The N2 and P3 activations in each experimental condition were presented in Figure 1.

### The correlations between behavioral performances and electrophysiological responses

We further analyzed the correlations between participants’ behavioral performances (reaction time, accuracy) and electrophysiological responses (peak latencies and amplitudes of N2 and P3) in the affective congruent conditions (HHHHH, NNNNN, SSSSS) and affective incongruent conditions (SSHSS, NNHNN, HHSHH, NNSNN, HHNHH, SSNSS). Participants’ response accuracy negatively correlated with N2 peak latency in the affective congruent condition (*r*= -0.5, *p*<0.05). Participants’ reaction time positively correlated with N2 peak latency in the affective congruent condition (*r*= 0.66, *p*<0.005) and the affective incongruent condition (*r*= 0.57, *p*<0.05), and the reaction time negatively correlated with P3 amplitude in the affective congruent condition (*r*= -0.63, *p*<0.01) and the affective incongruent condition (*r*= -0.6, *p*<0.01).

## Discussion

The current study investigated the temporal dynamic of cognitive control processing on affective conflicts. The important findings were observed during the affective conflict control processes: the affective incongruent condition (SSHSS) induced larger N2 amplitude and shorter P3 peak latency than the affective congruent condition (HHHHH), and the affective incongruent condition (HHSHH) elicited larger P3 amplitude than the congruent condition (SSSSS).

### Behavioral performances during the affective conflict control

The behavioral results showed that it took participants longer response time and lower accuracy to identify sad target expressions than happy and neutral target expressions, which was consistent with previous findings that individuals had slower response speed in the judgment of negative expressions than positive expressions [14]. More importantly, the significant flanker effect was also observed and participants exhibited faster response speed in identifying neutral target faces when they were flanked by neutral distractors than by happy distractors. These findings illustrated that there was response advance when the affective valence of targets was identical with the contexture information [5,7,15,16].

The current behavioral results might be explained by the distractor devaluation (DD) model which regarded that attentional inhibition processing on the distractors would affect the affective evaluation on the target [17], and positive distractors might attract more attention source and lead to harder inhibition control processing on them, therefore, it took participants longer time to evaluate the neutral target expression when they were flanked by happy distractors [5,6]. The current behavioral findings did not completely replicate Fenske & Eastwood’s study [5], and some flanker effects diminished in our current study, which might be due to that real human faces, compared with simple cartoon faces, drew more attention and left fewer attention resources for further conflict control processing.

### N2 responses for affective conflict monitoring

The N2 response was regarded as reflecting conflict monitoring [2], and the investigation of N2 responses during affective conflict control processing might also contribute to the understanding of cognitive control theories on emotion [18,19]. Our current study observed that the SSHSS condition induced larger N2 amplitude than the HHHHH condition. These results reflected that affective incongruent condition (higher conflict trials) induced stronger conflict monitoring processing with enhanced N2 amplitude than affective congruent condition (lower conflict trials) [9,10]. It was argued that participants’ visual field of view was widened by positive information when they executed both attention and emotion processes [20–22], therefore, it would be easier to detect the conflicts between happy target expression and its distractors. The current study also found that N2 activation induced in the SSHSS condition was stronger than that in the XXHXX condition, which illustrated that affective conflict monitoring processing was stronger than non-conflict condition.

Moreover, the current N2 activation was mainly distributed over the frontal and central areas, which suggested that neural function of monitoring processing on affect conflicts might be also relied on the frontal lobe, especially the ACC [1,2,4,23]. Chiew and Braver [24] adopted an emotional AX Continuous Performance Task (AX-CPT) to investigate the neural circuitry of emotional conflict control processing, in which the cue stimuli (“A”) offered a context for appropriate response selection to the subsequent probe (“X”), and it was reported that ACC and lateral prefrontal cortex (PFC) were activated during the affective conflict control processing.

### P3 responses for attention control on affective conflicts

It was currently observed that the affective incongruent condition SSHSS induced faster P3 response than the affective congruent HHHHH condition, and the flanker effect was also significant that the HHSHH condition induced larger P3 amplitude than the SSSSS condition. These results illustrated P3 responses could be induced not only in non-affective conflict control processes [13,25], but also during conflict control processes on affective information. The present findings also manifested that the affective incongruent trials induced larger P3 activation than the congruent trials [12,13,26]. In addition, the current P3 activation mainly reflected executive evaluation processing on facial expressions, and the neural distributions for affective attentional control were strongly related to central and central-parietal areas [11,27,28].

### N2 and P3 responses and affective conflict control

The N2 latency indexed brain’s processing speed of conflict detection [9,10], and the current correlation analysis findings indicated that participants’ better accuracy correlated with their faster neural processing of conflict detection [1,2,9]. It was also currently found that participants’ reaction time correlated with N2 latency and P3 amplitude, which might illustrate that individuals’ faster response of conflict control performances strongly related with their faster neural processing of conflict monitoring and the stronger neural activation during attentional control on the conflicts [9,10,13,26,29].

More importantly, it was currently revealed that the separate flanker effects were induced by positive and negative targets during the processing phase of attentional control: the flanker effects induced by positive targets related with the neural processing speed (peak latency) and the flanker effects induced by negative targets presented the extent of neural activation (peak amplitude). Taken together the findings of N2 and P3 responses, it was observed that the flanker effects induced by positive targets occurred at both conflict monitoring and attentional control processes, while the flanker effects induced by negative targets occurred only during attentional control processing.

## Conclusion

The current study testified the neural dynamic of affective conflict control on human facial expressions. By using ERP technique, the neural processes of conflict monitoring and attentional control were further subdivided, and the affective incongruent condition (SSHSS) induced larger N2 amplitude than the affective congruent condition (HHHHH) and neutral condition (XXHXX) during the conflict monitoring processing. During the attentional control processing, the affective incongruent condition (HHSHH) evoked larger P3 amplitude than the affective congruent condition (SSSSS). The experimental results supported our initial hypotheses, and the current findings also supported the cognitive control theory. This study further shed light on the idea that the interaction between attention and different emotion information (positive or negative) might act differently at different temporal courses.

## Materials and Methods

### Ethics Statement

This study was proved by the Ethics Committee of Institute of Psychology, Chinese Academy of Sciences. All the participants provided written informed consent prior to their participation.

### Participants

Eighteen right-handed participants (10 males, 8 female, ages from 22 to 31 years old, average 25.1 years old) were paid 100 RMB for participating in the current ERP experiment, and all of them reported normal or corrected-to-normal visual acuity and were naïve to the purpose of the experiment. None of them reported neurological or psychiatric problems.

### Materials

**Figure 2:**
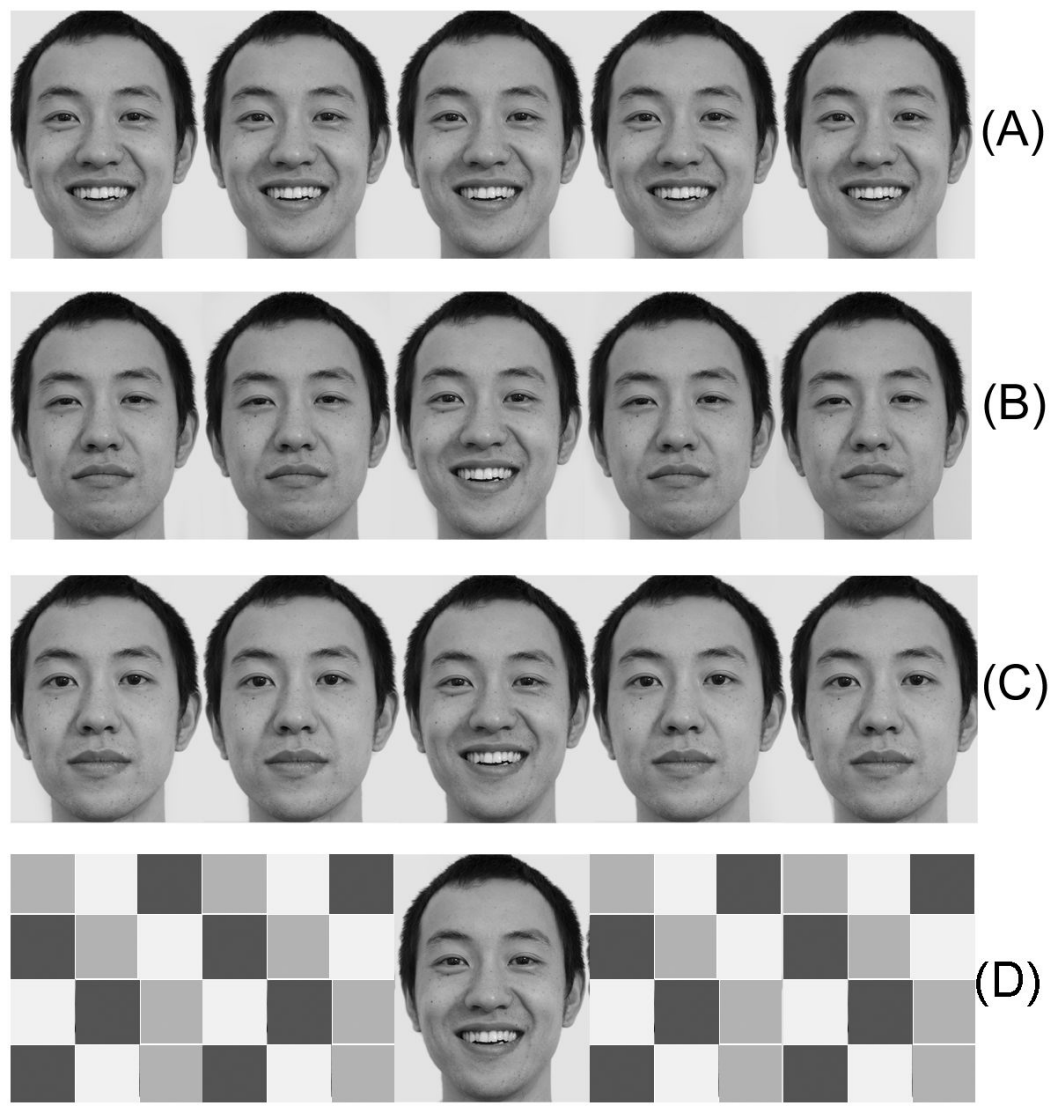
The stimulus samples (Due to the privacy rights, would the reader please note that the present pictures were not the stimuli used in the experiment The model in the sample pictures agreed to publish his pictures in the journal and his agreement file had been sent to the Journal Office of PLOS ONE.) Figure 2A showed the stimulus sample for HHHHH, Figure 2B for SSHSS, Figure 2C for NNHNN, and Figure 2D for XXHXX.

The facial expression stimuli were from 6 Chinese models (3 males and 3 females, ages from 25–29 years old). The central target expression was happy, sad or neutral expression with two distractor expressions (either happy, sad, neutral expressions, or scramble blocks) on the bilateral sides. Each stimulus adopted one model’s face. All the adopted stimuli were pre-evaluated by an independent group of 10 volunteers and 3 experts on the valence of the expressions, the arousal levels and intensity levels (inter-rater reliability: Cronbach’s α = 0.93). The prior evaluations showed that all the used stimulus expressions got the highest scores and were appropriate for the current study. Each stimulus was presented on a light grey screen of a 17-inch computer monitor (1024×768 at 100 Hz) with visual angle of 3.8° horizontally and 1° vertically. There were 3 types of experimental conditions: the affective congruent condition (3 kinds of stimuli: happy target expression-happy expression distractors [HHHHH], sad target-sad distractors [SSSSSS], neutral target -neutral distractors [NNNNN]), the affective incongruent condition (6 kinds of stimuli: happy target-sad distractors [SSHSS], happy target-neutral distractors [NNHNN], sad target-happy distractors [HHSHH], sad target-neutral distractors [NNSNN], neutral target-happy distractors [HHNHH], neutral target-sad distractors [SSNSS]), the neutral condition (3 kinds of stimuli: happy target flanked by scramble blocks [XXHXX], sad target flanked by scramble blocks [XXSXX], neutral target expression flanked by scramble blocks [XXNXX]). There were 24 trials in the practice section, 2 trials for each stimulus. There were 6 blocks in the formal experiment, and each block consisted of 180 trials (each stimulus was presented 15 times). Stimulus samples of HHHHH, SSHSS, NNHNN and XXHXX were presented in Figure 2, and the model in the samples has given written informed consent (as outlined in the PloS consent form) to publication of his photograph.

### Procedure

Participants were seated on a comfortable chair with a straight angle to the center of the computer monitor, and the viewing distance was 65 cm. At the beginning of each trial, there was a fixation ‘+’ for 300 ms, and each stimulus was displayed for 700 ms, followed by a blank screen for 500ms. The inter-stimulus interval was 2000 ms. Participants were required to concentrate on the target faces and to identify the categories of the expressions by pressing corresponding buttons (labeled with words “happy”, “sad”, “neutral”) as fast and correctly as possible. After each block, participants were allowed to have 2-3 minutes break. Participant’s index finger of left hand and index and middle fingers of right hand were required to press the buttons, and the usages of fingers for the three facial expressions were balanced among participants.

### ERP Recording and Data Analysis

Electroencephalograms (EEG) were recorded from 64 scalp electrodes embedded in a NeuroScan Quick-Cap. Electrode positions were placed according to 10-20 system locations, and four bipolar electrodes monitoring horizontal and vertical EOG (HEOG and VEOG) were positioned on the outer canthi of two eyes and in the inferior and superior areas of left eye, respectively. The skin resistance of each electrode was adjusted to less than 5 kΩ. EEG was continuously recorded at a sample rate of 1000 Hz using nose reference. The signal was amplified using Synamps 2 amplifiers and online band-pass filter at 0.05-100 Hz. We epoched the EEG signal with 100 ms prior to and 900 ms after the stimulus onset, and the pre-stimulus interval was used for baseline correction. Epochs contaminated by eye blinks, eye movements, or muscle potentials exceeding ±50 µV at any electrode were excluded from averaging. Overall, less than 10% of the epochs were excluded from further analysis. The trials were averaged for each experimental stimulus kind, and ERP signals were Zero Phase Shift filtered offline (bandpass: 0.1-30 Hz, slope: 24 dB/oct).

According to previous studies on conflict control [8–11], the peak amplitude and latency of N2 activation (at 150-350 ms range after stimulus onset) on the electrodes over the frontal and central areas (F3, FC3, Fz, FCz, F4, FC4) were recorded and further analyzed by repeated-measures ANOVAs for the conflict monitoring processing, and the peak amplitude and latency of P3 activation (at 300-600 ms range after the stimulus onset) on the electrodes over the central and parietal areas (C3, CP3, P3, Cz, CPz, Pz, C4, CP4, P4) were recorded and analyzed for attentional control processing. The Greenhouse corrections were applied to the results of ANOVA analyses. Significant main effects and interaction effects were further calculated by simple effect analyses and pair-wise comparisons (adjusted by Sidak test).
